# Arthroscopic Assessment of Temporomandibular Joint Pathologies—Is It Possible for Non-Specialists in Arthroscopy? Analysis of Variability and Reliability of Dental Students’ Ratings after a Comprehensive One-Semester Introduction

**DOI:** 10.3390/jcm13143995

**Published:** 2024-07-09

**Authors:** Lennard-Luca Brüning, Yannick Rösner, Axel Meisgeier, Andreas Neff

**Affiliations:** 1 Department of Oral and Craniomaxillofacial Surgery, UKGM GmbH, University Hospital Marburg, 35043 Marburg, Germany; 2 Faculty of Medicine, Philipps University, 35043 Marburg, Germany; 3 Faculty of Medicine, Heinrich-Heine Universität Düsseldorf, 40225 Duesseldorf, Germany

**Keywords:** TMDs, TMJ pathology, arthroscopic assessment, inter-rater reliability, rating-scale

## Abstract

**Background**: Arthroscopy of the temporomandibular joint (TMJ) plays a long-established role in the diagnostics and therapy of patients suffering from arthrogenic temporomandibular disorders (TMDs), which do not respond adequately to conservative/non-invasive therapy. However, the interpretation of arthroscopic findings remains challenging. This study investigates the reliability and variability of assessing arthroscopic views of pathologies in patients with TMDs by non-specialists in arthroscopy and whether a standardized assessment tool may improve correctness. **Methods**: Following a comprehensive one-semester lecture, dental students in the clinical stage of education were asked to rate 25 arthroscopic views (freeze images and corresponding video clips) regarding the severity of synovitis, adhesions, and degenerative changes on a scale of 0–10 (T1). The results were compared to ratings stated by two European-board-qualified academic OMF surgeons. In a second round (T2), the students were asked to repeat the ratings using a 10-point rating scheme. **Results**: With regard to all three subcategories, congruency with the surgeons’ results at T1 was at a low level (*p* < 0.05 in 19/75 cases) and even decreased at T2 after the implementation of the TMDs-SevS (*p* < 0.05 in 38/75 cases). For both T1 and T2, therefore, the inter-rater agreement was at a low level, showing only a slight agreement for all three subcategories (Fleiss’ Kappa (κ) between 0.014 and 0.099). **Conclusions**: The judgement of the arthroscopic pathologies of the TMJ remains an area of temporomandibular surgery that requires wide experience and training in TMDs to achieve expertise in TMJ arthroscopic assessments, which cannot be transferred by theoretical instruction alone.

## 1. Introduction

Temporomandibular disorders (TMDs) comprise a heterogenous group of musculoskeletal and neuromuscular diseases that can generally be divided into articular and non-articular disorders [1,2]. TMDs affect the masticatory system, as well as the temporomandibular joint and surrounding muscular and osseus structures [1,3,4]. 

The most common clinical symptoms are reduced mouth opening, eventually associated with joint sounds during motion and myogenic and/or arthrogenic pain [5,6,7]. Possible causes of TMDs include trauma, systemic diseases, iatrogenic as well as mental health disorders, and bruxism (clenching and grinding) [8,9,10]. Manifesting with a incidence of 34% in the world population and a prevalence of 29% in Europe [11], TMDs are the third most significant orofacial disorder, primarily affecting females aged between 20 and 40 [11,12,13].

Arthroscopy of the temporomandibular joint (TMJ), first performed by Ohnishi in 1975 [14], offers a valuable and secure diagnostic modality for patients presenting articular disorders which do not respond adequately to conservative therapeutic modalities [8,15,16]. Arthroscopy can also be complemented by adjunct therapeutic measures like arthrocentesis and/or intra-articular medication, exhibiting evidence-based efficacy in both diagnostic and therapeutic aspects of TMDs’ management [16,17,18].

Despite great progress in the minimally invasive arthroscopy of the TMJ, much uncertainty still exists about the correlation of pathologies observed in diagnostic arthroscopy, as well as clinical and histological findings [19]. One possible explanation might be founded in a demand of expert knowledge and proficiency to master the skill of temporomandibular joint arthroscopy. Therefore, incongruences in the interpretation of arthroscopic findings could potentially contribute to missing clinically relevant correlations with the patients’ symptoms.

To the best of our knowledge, no prior study has focused on the need of experience for the accurate interpretation of arthroscopic findings. Therefore, we decided to design a study to answer this open question.

Utilizing a cohort of dental students in the clinical stage of dental education, we administered a comprehensive one-semester lecture (i.e., appr. 50 lecture hours) on TMDs, including typical temporomandibular joint (TMJ) arthroscopy findings, followed by a comparative analysis of the students’ evaluation of arthroscopic pathologies against those of seasoned specialists.

To allow for a standardized assessment, we adapted the 11-point severity score published in 2001 by Segami and colleagues. This 11-point severity score describes synovitis, adhesion, and degenerative change as the primary sub-categories characterizing arthroscopic findings in patients with articular temporomandibular joint disorders (TMDs) [20].

As the professional license to practice in Germany (i.e., approbation for dental practitioners) usually does not implement the utilization and interpretation of arthroscopic techniques, it can therefore be argued that the students’ level of theoretical expertise in our cohort should be equal or most probably even exceed the usual knowledge of general dentists and/or average medical trainees in OMFS surgery, both of whom quite frequently remain without having more profound clinical experience in TMDs, unless attending to specialized postgraduate courses or being educated in specialized centers focusing on TMJ/TMDs.

Our hypothesis was that the proficient and consistent assessment of arthroscopic findings requires a substantial degree of clinical experience as a TMJ surgeon and is not achievable even after attending comprehensive specialized theoretical training and/or short-term clinical exposure, e.g., during an observership or during dental/medical studies. 

## 2. Materials and Methods

### 2.1. Study Design and Sample Description

This study was performed as a repeated cross-sectional study enrolling a sample of clinical dental students in a German University (i.e., dental students during the 6th to 9th clinical semesters with the regular study period consisting of 10 semesters). These semesters were selected because the preclinical degree was used as proof of a basic understanding of anatomy and the basics of orofacial medicine. Since lectures on TMDs were conducted on a rotational basis at the university, the comprehension on this specific topic was even across the cohort. The comparators were two European-board-qualified academic OMF surgeons, both experienced in TMJ arthroscopy. We created a sample of 25 sequential arthroscopic views from 25 TMDs patients randomly selected by the first and approved by the last author. The arthroscopic videos were collected between 2009 and 2021 during the clinical routine for the documentation of the individual pathologies and were cut to clips of approx. 30 s. of length (A.N.) (Video S1).

The investigation was conducted during the 2023 summer semester (i.e., term April to July 2023) after the ethical approval of the present project by the Institutional Ethics Committee (protocol code AZ:220/21, date of approval: 13 June 2022). All students gave their approval to take part in this study and all patients consented to the anonymized use of their data. The research adhered to the ethical principles as stated in the World Medical Association’s Declaration of Helsinki.

Clinical dental students meeting the following criteria were included: aged ≥18 years, German or foreign (non-native German) students with German C1 level proficiency, measured with the help of the Goethe Certificate [21], had successfully passed their preclinical exams (preclinical education in 2023 comprising 5 semesters), had registered and regularly participated (>85%) in an OMFS course in the 2023 summer term comprising appr. 50 lecture hours (i.e., 45 min, each) on TMDs and pain management, including lectures focused on minimally invasive TMJ surgery, as well as had agreed to participate in this study and to return the scoring survey, and completed both surveys (T1 and T2) of 25 arthroscopic case studies involving various patterns of TMJ pathology. 

The exclusion criteria were met if students had attended less than 85% of the lectures, suffered from mental or physical illness at the time of data acquisition, and/or if they disagreed to participate in the study. The sample size calculation using G-Power (Version 3.1.9.6) indicated that, for a paired-sample t-test with an effect size of 0.5, a minimum of 54 subjects were required to achieve a confidence level of 95% with the real value being within ±5% of the measured value in order to achieve a power value greater than 95% [22]. 

Throughout the one-semester OMFS lecture focused on TMDs and TMJ surgery, students received comprehensive instruction covering the anatomy and pathology of the TMJ and a broad overview about TMDs and pain management in general and the specialized field of arthroscopy in the context of TMDs and TMJ surgery (including arthroscopic views and video clips of typical TMJ pathologies). 

At the end of the semester, the students were tasked with the assessment of rating 25 sequential arthroscopic views and were asked to rate the severity of the categories of synovitis, adhesions, and degenerative changes using a quantifiable nominal analogue scale ranging from 0 and 10, respectively. Each video clip was presented twice (i.e., ca. 1 min in total per view). At this stage (T1), the students did not receive any additional information with regard to the structured assessment based on the TMDs’ severity score published by Segami and colleagues in 2001 [20], to be used later at T2. 

Following the initial round of data collection (T1), we implemented an instruction on the TMDs’ severity score (TMDs-SevS) according to Segami et al. [20] (Table 1), followed by a 3-day interval for the self-studying of this TMDs-SevS before entering a second analogous survey session (T2). In contrast to T1, the students at T2 now were able to follow a standardized assessment based on the TMDs-SevS [20]. The severity of each identified category, again, was rated using a quantifiable scale ranging from 0 to 10 with orientation vis a vis grading, according to Table 1. 

The rationale for the second rating session was to assess whether the utilization of a scale describing various pathologies would enhance the evaluation of non-arthroscopic specialists in terms of reliability and validity.

The instructions and survey were in German, and the TMDs-SevS according to Segami et al. [20] was translated into German by two of the authors as native German speakers (L-L.B. and A.N.).

### 2.2. Study Variables

The primary predictor variable was the introduction of the TMDs-SevS according to Segami et al. [20]. 

The main outcome variable was TMDs-SevS’ rating for synovitis, adhesion, and degenerative change (Table 1) in the 25 arthroscopic cases, describing the given conditions of the TMJ. For both rounds of data collection, this variable was recorded as binary based on a discrepancy among the average scores rated in comparison to the scores rated by the master examiners (i.e., yes [significant difference in rated scores] vs. no significance).

A second outcome variable was the development in the variability of the students’ ratings when comparing the first (T1) and the second round (T2) of data generation. 

Additional variables examined in this study were the demographic factors of age, gender, as well as the students’ semester. 

### 2.3. Data Collection and Statistical Analyses

Raw data were collected and recorded in a Microsoft Excel 2019 document (Microsoft Corporation, Redmond, WA, USA) by the primary author (L-L.B.). Statistical analyses were then performed using RStudio (Posit team (2023). RStudio (Version 2023.09.01): Integrated Development Environment for R. Posit Software, PBC, Boston, MA. URL: http://www.posit.co/). An initial power analysis was performed using G-Power [22]. 

The Shapiro–Wilk and Kolmogorov–Smirnov tests were used to evaluate the normal distribution of the data before entering the computation using descriptive, bivariate, and multiple logistic regression statistics.

The testing of variability in terms of the inter-rater reliability was carried out with the help of Fleiss’ Kappa for both T1 and T2. For each subcategory, calculations were performed individually. The interpretation of Kappa was carried out as described by Landis and Koch: poor agreement: <0.00, slight agreement: 0.00–0.20, fair agreement: 0.21–0.40, moderate agreement: 0.41–0.60, substantial agreement: 0.61–0.80, and excellent agreement: 0.81–1.00 [23]. 

## 3. Results

This study cohort comprised 95 students. The educational backgrounds of these students were not distributed evenly (i.e., 17 students in their 6th semester (Auscultando), 20 students in their 7th semester (Practicando I), 30 in their eighth semester (Practicando II), 23 in their ninth semester (Practicando III), and 5 Erasmus exchange students). The master examiner group consisted of two European-board-qualified (FEBOMFS) academic OMF surgeons (>30 years of professional experience and >15 years, respectively). The average age of participating students was 24.5 ± 4.0 years with an age range between 21 and 48 years. Among the participants, 51 (53.7%) were females (Table 2). 

In each evaluation round (T1 and T2), each participant assessed three pathological conditions (NAS ratings for synovitis, adhesion, and degenerative changes each) for a total of 25 cases, thereby generating a dataset consisting of two times 75 data points for TMDs’ severity grading. The dataset, therefore, included 75 assessments conducted at T1 without the utilization of the TMDs-SevS according to Segami et al. [20], a second 75-data-point assessment conducted at T2 with the use of the TMDs-SevS, and another 75 assessments provided by the master examiners, also using the SevS as a golden standard. 

As the formal normality tests using Shapiro–Wilk and Kolmogorov–Smirnov tests did not confirm the normal distribution of the data, further statistical analyses were performed with non-parametric statistics. Fischer’s exact test was performed for the assessment of differences in the ratings between students and master examiners as expected frequencies for more than 20% of the severity grades were <5 and no normal distribution was found [24,25]. Age and gender were not statistically significant confounders. 

### 3.1. Comparison of T1 with Masters’ Ratings

A comparative analysis of data obtained during the initial data collection phase (T1) and masters’ assessments revealed significant differences in all three subcategories. These differences were found to be statistically significant (*p* < 0.05) in 6 of 25 cases for synovitis, 8 of 25 cases of adhesion, and 5 out of 25 cases concerning degenerative changes (Table 3, Table 4 and Table 5). Significant differences are marked within the tables with an asterisk. 

### 3.2. Comparison of T2 with Experts’ Ratings

Following the implementation of the TMDs-SevS according to Segami et al. [20], there was even a notable increase at T2 concerning the number of cases with significant differences (*p* < 0.05, Fischer’s Exact Test) in all the given subcategories. For synovitis, 13 out of the 25 cases showed a significant difference in comparison to the master examiners’ ratings (Table 6). For adhesion, 10 of 25 cases displayed statistically significant differences (Table 7) and 15 out of 25 cases for degenerative changes (Table 8). These significant differences are indicated within the respective tables.

### 3.3. Comparison of T1 and T2 with Regard to Master Examiners’ Ratings on an Individual Basis

**Figure 1:**
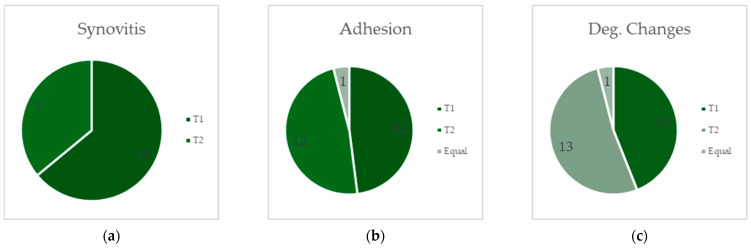
Figures (**a**–**c**) show the distribution of correct and close-to-correct (±1) ratings at T1 (darker grey) vs. T2 (medium grey) and equal ratings (light grey), with the master examiners’ ratings serving as reference; (**a**) refers to synovitis, (**b**) refers to adhesion, and (**c**) to degenerative changes.

When comparing the students’ ratings for T1 and T2 concerning the master examiners’ agreement, we decided to count as “correct” the students’ votes which were within ±1 of the master examiners’ ratings. There were 95 students’ ratings per view. Table 9 demonstrates the results for each subcategory. For synovitis, 16 out of 25 cases showed a higher number of agreements for T1 and only 9/25 for T2. In the subcategory of adhesion, 12/25 cases exhibited a higher number of agreements at both T1 and T2, with one case showing the same number of agreements. Regarding degenerative changes, we found 11/25 cases for T1 with a higher number of agreements, slightly increasing to 13 out of 25 cases for T2. One case displayed the same number of students agreeing. In total, there were 39 out of 75 cases with a higher number of correct ratings for T1, 34 out of 75 cases with a higher number of correct ratings for T2, and 2/75 cases with an equal count of correct ratings. These results are demonstrated in Figure 1.

The total amount of correct judgements was different within the three subcategories: Out of a total of 2375 ratings per subcategory (25 times 95 judgements each), we found 911 (38.4%) correct ratings for T1 and 815 (34.3%) for T2. For adhesion, there were 618 (26%) correct ratings for T1 and 684 (28.8%) for T2. Concerning degenerative changes, the number of correct ratings was at 552 for T1 (23.2%) and at 546 (23%) for T2 (Table 10). These differences were statistically significant at *p* > 0.01 for the categories of synovitis (*p*-value: 0.0038) and adhesion (*p*-value: 0.0032). For the category of degenerative changes and the overall number of correct ratings, the difference was found not to be statistically significant (*p*-value: 0.8363 and *p*-value: 0.0584, respectively).

### 3.4. Comparison of T1 and T2 of Student Cohorts’ with Master Examiners’ Results

When conducting a comparative analysis between the T1 and T2 results and setting them against the assessments of the master examiners, a notable decrease in alignment with the master examiners’ opinions became evident following the implementation of the TMDs-SevS according to Segami et al. [20] (Table 11). In all three subcategories, we observed an increase in the number of cases with significant differences. 

The most substantial increase was observed within the subcategory of degenerative changes where the instances of significant differences increased three-fold (from 5/25 to 15/25, *p*-value: 0.0004, Chi Square test). With regard to synovitis, the frequency of significant differences between T1 and T2 more than doubled (from 6/25 to 13/25; *p*-value: 0.0414, Chi Squar -test). Adhesion was the only category which exhibited just a modest increase in the total amount of significant differences (from 8/25 to 10/25; *p*-value: 0.5557, Chi Square test).

### 3.5. Inter-Rater Agreement at T1 and T2

In terms of the variability observed in the students’ rating, our findings revealed a notably low level of agreement, characterized by only a slight agreement according to Fleiss’ Kappa ((κ) < 0.2) [23]. 

During the initial round of data collection (T1), the Kappa (κ) values were 0.061 for synovitis, 0.032 for adhesion, and 0.014 for degenerative changes. With the inclusion of the SevS according to Segami et al. [20], TMDs’ Kappa (κ) values at T2 were calculated as 0.099 for synovitis, 0.07 for adhesion, and 0.03 for degenerative changes (Table 12).

Despite an improvement in agreement observed at T2 as compared to T1, it is noteworthy that there was no essential change with regard to the interpretation of the calculated Kappa values (κ). 

For the inter-rater agreement for the master examiner cohort, the overall agreement observed was found to be at a moderate level only, ranging from 0.5 for adhesion to 0.575 for synovitis and 0.577 for degenerative changes [23].

The developments in the inter-rater agreement could not be shown to be statistically significant between T1 and T2, but displayed statistically significant differences for the comparison of T1 and T2 against the master examiners’ inter-rater agreement (Table 13).

## 4. Discussion

Arthroscopy plays an important and well-established role in both the diagnostics and treatment of patients suffering from TMDs [16,17,26]. However, achieving proficiency in performing and interpreting arthroscopy of the temporomandibular joint seems to be an objective yet to be overcome. Classical teaching methods following the scheme of “observing, practicing, teaching”, where surgical skills are acquired under the supervision of experienced surgeons, seem to be insufficient when applied to arthroscopy [26,27]. 

This hypothesis is supported by the results of this study. Looking at the results for T1, the number of discrepancies between the ratings of the students’ cohort and those of master examiners is relatively high, occurring in twenty percent of cases for degenerative change, twenty-four percent of cases for synovitis, and even thirty-two percent of cases for adhesion. This discrepancy in the results fits into the low inter-rater agreement rates, undermining a high degree of uncertainty in the students’ judgement across all three subcategories. The lowest agreement rate was found for degenerative changes, despite being the subcategory with the lowest number of ratings with significant differences when compared to the master examiners’ results. This might be due to a high dispersion of ratings, resulting in a relatively high standard deviation. Analyzing the results on an individual basis, a similar pattern can be found. Out of the total of 2375 ratings assessed at T1, only 911 (38.4%) ratings were correct students’ ratings for synovitis. Concerning adhesion, it was even less with 618 (26%) correct ratings. Degenerative changes had the lowest number of correct ratings with only 552 (23.2%). Therefore, it can be argued that synovitis was the category least difficult to judge, whereas the correct judgement of degenerative changes posed a bigger challenge. 

Following the implementation of the TMDs-SevS according to Segami et al. [20], for T2, the number of cohort ratings with significant differences in comparison to the masters’ evaluations even increased across all three subcategories. This finding was contrary to our expectation. A potential explanation for the observed decline in rating-quality might be, that the rating scheme made the students more aware of the complexity of arthroscopic assessments, leading to a possibly more conservative rating as students attempted to consider various nuances. On an individual basis, the differences were not as pronounced as cohorts’ results would suggest. Despite the fact, that the number of cases with significant differences for synovitis more than doubled at the cohorts’ results, the total development in number of correct judgements was relatively small, decreasing only from 911 to 815 (4.1%). Regarding adhesion, the total number of correct judgements even increased on an individual basis. Though we found an increase of cases with significant differences of cohorts’ ratings for degenerative changes of 200%, the number of correct ratings on an individual basis did not change much and decreased by only 6 votes (0.2%). This discrepancy may be due to a reduction in correct judgements on an individual basis by a significant number of ratings for some specific cases, resulting in a decrease of rating quality of the cohort for these cases. For other cases the number of correct judgements increased for T2 without having an impact on the comparison of cohorts’ rating with regard to the masters’ ratings. When looking at the cases where a significant difference at T2 was present, it becomes clear that most of these cases were judged with extreme scores (e.g., very high (8–10) or very low (0–2) on the severity scale) by the master examiners. This suggests, that when implementing the TMDs-SevS [20], students’ ratings became less extreme when compared to T1. Ratings were somewhat more conservative, choosing a score in the middle rather than extremes values, i.e., the students exhibited a typical central tendency, well known from test psychology [28]. It is therefore possible, that the description of various pathologies distracts from the actual severity of pathologic findings inside the joint or that the TMDs-SevS according to Segami et al. [20] has made students more insecure about their ratings.

Fleiss’ Kappa showed a slight improvement for all three subcategories, even if not statistically significant, (Table 9) and descriptively, a reduction in standard deviation became evident. Therefore, it can be argued that the implementation of the TMDs-SevS [20] resulted in a higher consensus among students, albeit at a relatively low level with only a slight agreement, as described by Landis and Koch [23]. Still, the quality of judgment appeared to decrease for the overall cohort. One possible reason might be that “better” ratings in terms of a lower number of clear significant differences compared to the master examiners’ assessments at T1, resulted from the high discrepancy of students’ ratings. As the inter-rater agreement increased, the frequency of significant differences also increased, exposing a generally high insecurity in students’ ratings. 

Despite at a low level, at least a slight, though statistically not significant increase in the agreement after implementation of the TMDs-SevS was notable. This indicates that the implementation of a rating scheme basically might be a beneficial tool for (standardized) ratings of arthroscopy. In this context, it needs to be taken into account, that the severity scale presented by Segami and colleagues [20] did not follow the aim to offer a scale for assessing or generalizing arthroscopic findings, but rather to describe various pathologies and rank them according to their severity. One possibility to improve assessment of arthroscopy, therefore, would be to implement a scheme which focusses on different therapeutical treatment modalities resulting out of findings during arthroscopy of the temporomandibular joint. Such a therapeutical rating scheme, therefore, might be established based on the treatment modalities indicated for certain pathologies rather than on predefined categories regardless of the resulting treatment strategies. 

A high inter-rater agreement when assessing findings in arthroscopy is of paramount importance to allow for a standardizable treatment strategy for patients affected with TMDs. This statement is undermined by results of Al-Moraissi and colleagues when assessing different treatment modalities of arthrogenous TMDs [29,30,31], highlighting the existing challenge of implementing best fitting therapeutic recommendations. Inaccurate assessments of arthroscopic findings, therefore, may lead to mistakes in treatment planning as well as outcomes of various treatment modalities. Generating a reliable and reproducible evaluation of arthroscopic findings, therefore, plays a crucial role in establishing said therapeutic recommendations, potentially preventing overtreatment.

To the best of our knowledge no previous study was performed to assess inter-rater reliability for temporomandibular joint arthroscopy. Nevertheless, it was assessed for arthroscopy of other joints. In line with our findings, inter-rater reliability for the arthroscopic classification of hip pathology remained at a rather low level, even when performed by experienced surgeons [32,33]. Kappa (κ)-values of the expert examiners in our study were at a similar level as inter-rater reliability described in these studies. Nevertheless, our cohort of master examiners was relatively small, as we focused on non-experts (such as observers, i.e., those without the possibility to actively participate in and/or practice arthroscopy). Nevertheless, substantial differences for a single judgement might highly impact the Kappa (κ) for the given subcategory. It could, therefore, be useful to implement a follow-up study to assess the inter-rater reliability of a bigger number of Oral and Maxillofacial TMJ surgeons, thereby testing the inter-rater agreement among trainees and also experienced arthroscopic surgeons. Generally, it can be assumed that the difficulties in assessing arthroscopic views are not solely dependent on the severity of pathologies found inside the joint during arthroscopy, but rather on the evaluation of arthroscopic views in general, i.e., it is largely experience dependent.

Possible limitations to our study are choosing a cross-sectional study design, a limited length, as well as ratings being solely based on arthroscopic findings without the consideration of relevant practical information such as haptic feedback usually felt during arthroscopy nor information regarding the patient’s history, clinical symptoms or the outcomes of other diagnostic methods, usually performed prior to arthroscopy and known by the executing surgeon. 

As stated by previous studies, attaining skill and expertise in temporomandibular joint arthroscopy poses a great challenge [26]. The results of this study suggest that the said challenge was not only due to the complexity of the physical execution of arthroscopy, but additionally aggravated by obstacles in the correct assessment of arthroscopic findings. 

It should, therefore, be mentioned that established rating systems for TMDs such as the DC-TMDs [34] are mainly based on the patient’s clinical symptoms and/or radiologic findings (i.e., MRI). Arthroscopy, therefore, serves more as a diagnostic and/or therapeutic tool rather than as a sole basis for assessing the various degrees or stages of TMDs’ expression [2,34,35]. A prospective follow-up study, including the integration of MRI findings and/or patients’ clinical history, could possibly address this limitation and might, therefore, allow for an improvement in reliability and variability when rating arthroscopic views, as well as enhancing the transferability of this study’s findings into clinical practice. Particularly for assessing the severity of synovitis, taking the patient’s medical history and clinical findings into account could result in an improvement, as several studies have demonstrated a correlation between the degree of synovitis and pain [7,36,37].

Regarding the arthroscopy videos utilized, it is important to note that the clips were of approximately 30 s in length and were only presented twice during the assessment. Students, therefore, only had a limited amount of time for the assessment of the demonstrated condition within the joint, and the videos presented typical findings, but not necessarily a holistic view of the joint. In clinical practice, a TMJ surgeon would have more time to assess the different recesses of the upper and potentially even the lower joint compartment when performing arthroscopy. The raters participating in the study were dependent solely on the views provided. There was no option of adjusting viewing angles or to focus on different regions of the joint. Nevertheless, a general overview of each joint was provided, and the given limitation was kept in mind when the specific videos were generated. Additionally, it can be noted that the master examiners’ judgments were based on the same videos, allowing for a general comparison of the results, although even the master examiners, themselves, were at a moderate level of congruency only. 

As already mentioned, a lack of haptic feedback, a feature surgeons usually have when actually performing arthroscopy, might have an impact on assessing intra-articular pathologies and limits the transferability of our results into the clinical practice [32]. As a consequence of this lack of haptic feedback usually provided during arthroscopy, the raters were not able to judge, e.g., limitations of joint mobility.

Choosing a repeated cross-sectional study design also brings limitations, such as the risk of having confounding factors. It is likely that the students spent different amounts of time in order to get familiar with the TMDs-SevS according to Segami et al. [20]. This might have had an impact especially on the results generated at T2. 

Based on the findings of this study, it is evident that arthroscopy of the temporomandibular joint is a highly specialized field within OMF Surgery that demands extensive expertise and training. This study points out the difficulties of dental students—even though well trained in the theoretical background of TMDs—in assessing such arthroscopic views, stressing a need of expertise that surpasses the clinical stage of theoretical university education. Nevertheless, our dental students, having received a 50 h lecture on TMDs should be well on a par with the theoretical knowledge of TMJ pathologies found among, e.g., general practitioners or younger trainees in OMFS. This is also underlined by the conspicuous lack of differences between the student cohorts, i.e., the theoretical training may be supposed to have offered a comparable theoretical background on TMJ pathologies for all participating students. The implementation of a rating scheme still to be developed can basically be suggested to reduce variability in the evaluation of different arthroscopic views. Still, such a rating scheme should take different diagnostical options into consideration, thereby allowing optimized treatment decisions, rather than being solely based on the graded description of pathological findings within the joint. In addition, future studies assessing different potential rating schemes should take into account the difficulties for non-experts in assessing arthroscopic views, therefore involving multiple TMJ surgeons with different levels of expertise, and assessing the impact on clinical decision making.

## 5. Conclusions

This study highlights the difficulties of arthroscopic assessments in the temporomandibular joint, the need for expertise, but also suggests the general potential of utilizing a rating scheme for arthroscopy of the TMJ to improve reliability in ratings and therefore potentially optimizing therapeutic outcomes. 

Last but not least, this study also undermines the importance of continued training and hands-on experience in arthroscopy within the field of Oral and Maxillofacial Surgery.

## Figures and Tables

**Table 1:** TMDs-SevS according to Segami et al. [20].

Score	Synovitis	Adhesion	Degenerative Changes
0	Normal pale, almost translucent, synovial lining with a fine network of anastomosing small blood vessels	No adhesion or fibrous change	Normal articular cartilage
1	Increased vascularity and capillary hyperemia (mild)	Filmy adhesion (mild)	Softening in probe palpation (mild)
2	Increased vascularity and capillary hyperemia (moderate)	Filmy adhesion (moderate-to-severe)	Softening in probe palpation (moderate-to-severe)
3	Increased vascularity and capillary hyperemia (severe)	Fibrosynovial band (mild to moderate)	Convex–concave surface
4	Capillary dilatation and increasing network (mild-to-moderate)	Fibrosynovial band (severe)	Fibrillation (mild)
5	Capillary dilatation and increasing network (severe)	Fibrous band (mild-to-moderate)	Fibrillation (moderate)
6	Contact bleeding occurs on probe palpation (mild-to-moderate)	Fibrous band (severe)	Fibrillation (severe)
7	Contact bleeding occurs on probe palpation (severe)	Pseudowall formation (mild-to-moderate)	Bone exposure (mild)
8	Microbleeding and effusion	Pseudowall formation (severe)	Bone exposure (moderate)
9	Granulative change, effusion, and debris (mild-to-moderate)	Capsular fibrosis (mild-to-moderate)	Bone exposure (severe)
10	Granulative change, effusion, and debris (severe)	Capsular fibrosis (severe)	Intracapsular fibrosis

**Table 2:** Descriptive analysis of the study sample.

	Total	6th Semester	7th Semester	8th Semester	9th Semester	Erasmus
Count	95	17	20	30	23	5
Age ± Standard deviation	24.57 ± 4.08	23 ± 3.46	25.5 ± 5.94	24.3 ± 3.81	25.26 ± 2.36	23.6 ± 3.65
Female (%)	51 (53.7%)	9 (52.9%)	9 (45%)	20 (66.7%)	13 (56.5%)	4 (80%)
Male (%)	43 (46.3%)	8 (47.1%)	11 (55%)	10 (33.3%)	10 (43.5%)	1 (20%)

**Table 3:** Comparison of students’ and master examiners’ rating with Fisher’s Exact Test for Synovitis (T1).

Synovitis Case	Students’ Rating (Mean ± SD)	Masters’ Rating (Mean ± SD)	*p*-Value (Fisher’s Exact Test)
1	3.64 ± 1.63	3.0 ± 0	0.52
2	3.38 ± 2.02	1.0 ± 0	0.16
3	7.05 ± 2.01	5.0 ± 0	0.07
4	6.16 ± 2.31	4.0 ± 0	0.16
5	2.86 ± 1.8	1.5 ± 0.7	1
6	3.33 ± 2.1	9.0 ± 0	0.0026 **
7	7.72 ± 1.73	4.5 ± 0.7	0.017 *
8	4.51 ± 1.84	2.5 ± 0.7	0.5
9	2.26 ± 1.57	4.0 ± 0	0.04
10	3.4 ± 1.96	3.0 ± 0	0.23
11	5.85 ± 1.66	5.0 ± 0	0.35
12	3.48 ± 1.96	5.5 ± 6.4	0.008 **
13	2.97 ± 1.56	2.0 ± 0	0.41
14	6.74 ± 2.35	4.5 ± 0.7	0.59
15	6.52 ± 1.86	5.0 ± 0	0.24
16	5.61 ± 1.68	4.0 ± 0	0.35
17	6.42 ± 1.95	5.0 ± 0	0.78
18	8.06 ± 2.16	9.0 ± 0	0.55
19	6.29 ± 1.89	5.0 ± 0	0.19
20	6.98 ± 1.98	10.0 ± 0	0.02 *
21	3.06 ± 1.91	0.5 ± 0.7	0.27
22	2.98 ± 1.78	4.5 ± 0.7	0.30
23	4.94 ± 1.87	7.0 ± 4.2	0.03 *
24	4.8 ± 1.94	7.0 ± 0	0.16
25	6.22 ± 1.78	9.0 ± 1.4	0.07

* *p*-Value < 0.05; ** *p*-Value < 0.005.

**Table 4:** Comparison of students’ and master examiners’ rating with Fisher’s Exact Test for adhesion (T1).

Adhesion Case	Students’ Rating (Mean ± SD)	Masters’ Rating (Mean ± SD)	*p*-Value (Fisher’s Exact Test)
1	5.85 ± 2.36	3.0 ± 0	0.38
2	5.87 ± 2.45	5.5 ± 0.7	0.8
3	4.55 ± 2.33	2.0 ± 0	0.12
4	2.07 ± 2.02	0	0.55
5	6.44 ± 2.39	4.0 ± 0	0.2
6	4.95 ± 2.3	0	0.005 **
7	3.77 ± 2.34	4.0 ± 0	0.61
8	6.68 ± 2.28	0	0.02 *
9	4.53 ± 2.2	0	0.01 *
10	5.24 ± 2.52	0	0.008 **
11	5.62 ± 2.48	4.5 ± 0.7	1
12	6.67 ± 2.29	6.0 ± 0	0.5
13	4.17 ± 2	3.0 ± 4.2	0.12
14	2.86 ± 2.19	7.0 ± 0	0.03 *
15	2.24 ± 2.3	6.0 ± 1.4	0.09
16	5.91 ± 2.46	0	0.02 *
17	4.11 ± 2.03	6.0 ± 0	0.39
18	7.2 ± 2.12	3.5 ± 0.7	0.03 *
19	2.94 ± 1.99	0.5 ± 0.7	0.46
20	6.19 ± 2.02	5.5 ± 2.1	0.58
21	5.12 ± 2.17	0	0.008 **
22	2.18 ± 1.87	0	0.33
23	5.67 ± 2.04	4.0 ± 1.4	0.41
24	4.18 ± 2.03	4.5 ± 0.7	0.92
25	5.16 ± 2.34	7.5 ± 2.1	0.27

* *p*-Value < 0.05; ** *p*-Value < 0.005.

**Table 5:** Comparison of students’ and master examiners’ rating with Fisher’s Exact Test for degenerative changes (T1).

Degenerative Changes Case	Students’ Rating (Mean ± SD)	Masters’ Rating (Mean ± SD)	*p*-Value (Fisher’s Exact Test)
1	4.15 ± 2.99	5.0 ± 0	0.02 *
2	3.37 ± 2.77	4.0 ± 0	0.41
3	4.18 ± 2.75	4.0 ± 0	0.10
4	2.84 ± 2.38	0	0.75
5	3.85 ± 2.34	7.0 ± 0	0.11
6	3.67 ± 2.65	4.5 ± 0.7	1
7	3.59 ± 2.63	4.5 ± 0.7	0.54
8	4.55 ± 2.71	4.5 ± 0.7	1
9	3.27 ± 2.43	7.0 ± 0	0.07
10	3.87 ± 2.69	4.5 ± 0.7	0.28
11	4.34 ± 2.37	4.5 ± 0.7	0.87
12	4.82 ± 2.68	8.5 ± 0.7	0.37
13	3.09 ± 2.42	6.5 ± 3.5	0.09
14	2.87 ± 2.26	0	0.75
15	2.7 ± 2.09	0	0.29
16	2.61 ± 2.46	6.5 ± 0.7	0.09
17	2.51 ± 2.27	7.5 ± 0.7	0.01 *
18	7.04 ± 2.32	10.0 ± 0	0.19
19	3.74 ± 2.68	0	0.46
20	5.7 ± 2.72	5.0 ± 0	0.29
21	4.38 ± 2.79	8.0 ± 0	0.02 *
22	2.29 ± 2.17	0	0.81
23	5.63 ± 2.18	9.0 ± 0.7	0.1
24	4.59 ± 2.34	2.0 ± 0	0.03 *
25	5.19 ± 2.81	10.0 ± 0	0.003 *

* *p*-Value < 0.05.

**Table 6:** Comparison of students’ and master examiners’ rating with Fisher’s Exact Test for synovitis (T2).

Synovitis Case	Students’ Rating (Mean ± SD)	Masters’ Rating (Mean ± SD)	*p*-Value (Fisher’s Exact Test)
1	4.04 ± 1.93	5.0 ± 0	0.13
2	5.55 ± 1.99	4.0 ± 0	0.0006 ***
3	3.95 ± 1.21	4.0 ± 0	0.76
4	2.37 ± 1.54	0	0.14
5	2.67 ± 1.92	7.0 ± 0	1
6	3.62 ± 1.75	4.5 ± 0.7	0.0004 ***
7	6.74 ± 1.34	4.5 ± 0.7	0.02 *
8	6.56 ± 2.18	4.5 ± 0.7	0.02 *
9	4.41 ± 1.3	7.0 ± 0	0.46
10	2.66 ± 2.07	4.5 ± 0.7	0.19
11	4.73 ± 1.27	4.5 ± 0.7	1
12	4.81 ± 1.96	8.5 ± 0.7	0.006 **
13	1.21 ± 0.94	6.5 ± 3.5	0.17
14	7.58 ± 1.35	0	0.003 **
15	2.47 ± 1.24	0	0.02 *
16	3.6 ± 2.6	6.5 ± 0.7	0.23
17	6.5 ± 2.74	7.5 ± 0.7	0.5
18	5.37 ± 1.92	10.0 ± 0	0.06
19	1.35 ± 1.25	0	0.005 **
20	5.32 ± 2.34	5.0 ± 0	0.0006 ***
21	3.6 ± 1.59	8.0 ± 0	0.009 **
22	6.01 ± 1.74	0	0.25
23	4.43 ± 2.84	9.0 ± 0.7	0.003 **
24	1.8 ± 1.26	2.0 ± 0	0.0004 ***
25	3 ± 2.15	10.0 ± 0	0.002 **

* *p*-Value < 0.05; ** *p*-Value < 0.005; *** *p*-Value < 0.0005.

**Table 7:** Comparison of students’ and master examiners’ rating with Fisher’s Exact Test for adhesion (T2).

Adhesion Case	Students’ Rating (Mean ± SD)	Masters’ Rating (Mean ± SD)	*p*-Value (Fisher’s Exact Test)
1	4.61 ± 0.53	5.0 ± 0	0.46
2	6.18 ± 1.52	4.0 ± 0	1
3	2.62 ± 2.53	4.0 ± 0	0.16
4	5.73 ± 1.72	0	0.001 **
5	5.21 ± 1.77	7.0 ± 0	0.29
6	1.58 ± 1.53	4.5 ± 0.7	0.11
7	3.39 ± 1.68	4.5 ± 0.7	0.05
8	5.39 ± 2.18	4.5 ± 0.7	0.0002 ***
9	4.55 ± 2.08	7.0 ± 0	0.03 *
10	4.1 ± 2.2	4.5 ± 0.7	0.02 *
11	1.35 ± 1.58	4.5 ± 0.7	0.02 *
12	5.11 ± 1.75	8.5 ± 0.7	0.63
13	3.71 ± 1.88	6.5 ± 3.5	0.08
14	5.07 ± 2.15	0	0.38
15	5.37 ± 2.51	0	0.44
16	6.15 ± 1.68	6.5 ± 0.7	0.0004 ***
17	2.34 ± 2.05	7.5 ± 0.7	0.04 *
18	4.2 ± 1.91	10.0 ± 0	0.75
19	6.37 ± 1.71	0	0.001 **
20	2.84 ± 1.82	5.0 ± 0	0.1
21	5.49 ± 1.82	8.0 ± 0	0.0002 ***
22	4.47 ± 2.17	0	0.001 **
23	4.14 ± 2.72	9.0 ± 0.7	0.61
24	3.33 ± 1.71	2.0 ± 0	0.61
25	4.49 ± 2.03	10.0 ± 0	0.17

* *p*-Value < 0.05; ** *p*-Value < 0.005; *** *p*-Value < 0.0005.

**Table 8:** Comparison of students’ and master examiners’ rating with Fisher’s Exact Test for degenerative changes.

Degenerative Changes Case	Students’ Rating (Mean ± SD)	Masters’ Rating (Mean ± SD)	*p*-Value (Fisher’s Exact Test)
1	4.32 ± 1.82	5.0 ± 0	0.3
2	4.49 ± 1.97	4.0 ± 0	0.48
3	2.61 ± 1.79	4.0 ± 0	0.32
4	4.41 ± 2.18	0	0.004 **
5	3.68 ± 1.85	7.0 ± 0	0.002 **
6	2.21 ± 1.74	4.5 ± 0.7	0.01 *
7	4.53 ± 1.87	4.5 ± 0.7	1
8	5.09 ± 1.69	4.5 ± 0.7	0.88
9	3.64 ± 1.75	7.0 ± 0	0.003 **
10	3.43 ± 2.42	4.5 ± 0.7	0.29
11	1.94 ± 1.98	4.5 ± 0.7	0.05
12	4.51 ± 2.17	8.5 ± 0.7	0.04 *
13	3.8 ± 2.14	6.5 ± 3.5	0.01 *
14	5.48 ± 1.83	0	0.01 *
15	5.28 ± 2.32	0	0.005 **
16	5.48 ± 1.98	6.5 ± 0.7	0.75
17	3.66 ± 2.4	7.5 ± 0.7	0.16
18	3.35 ± 1.64	10.0 ± 0	0.0006 ***
19	4.34 ± 2.39	0	0.005 **
20	3.34 ± 2.23	5.0 ± 0	0.02 *
21	4.26 ± 2.37	8.0 ± 0	0.0004 ***
22	4.19 ± 1.79	0	0.0004 ***
23	4.34 ± 2.55	9.0 ± 0.7	0.02 *
24	3.97 ± 2.05	2.0 ± 0	0.12
25	4.99 ± 1.78	10.0 ± 0	0.02 *

* *p*-Value < 0.05; ** *p*-Value < 0.005; *** *p*-Value < 0.0005.

**Table 9:** Number of students (% out of 95 participants) within ±1 range of masters’ ratings for each video.

Case	Synovitis T1	Synovitis T2	Adhesion T1	Adhesion T2	Deg. Changes T1	Deg. Changes T2
1	58 (61.1%)	42 (44.2%)	27 (28.4%)	47 (49.5%)	27 (28.4%)	49 (51.6%)
2	32 (33.7%)	9 (12%)	46 (48.4%)	71 (74.7%)	9 (12%)	14 (14.7%)
3	25 (26.3%)	67 (70.1%)	32 (33.7%)	55 (57.9%)	17 (17.9%)	21 (22.1%)
4	27 (28.4%)	37 (38.9%)	49 (51.6%)	1 (1.1%)	35 (36.8%)	4 (4.2%)
5	48 (50.5%)	59 (62.1%)	26 (27.4%)	50 (52.7%)	15 (15.8%)	21 (22.1%)
6	5 (5.2%)	2 (2.1%)	10 (10.5%)	65 (68.4%)	28 (29.5%)	7 (7.4%)
7	11 (11.6%)	9 (12%)	36 (37.9%)	44 (46.3%)	19 (20%)	43 (45.3%)
8	28 (29.5%)	8 (8.4%)	4 (4.2%)	3 (3.2%)	31 (32.6%)	41 (43.2%)
9	23 (24.2%)	72 (75.8%)	8 (8.4%)	8 (8.4%)	14 (14.7%)	17 (17.9%)
10	51 (53.7%)	40 (42.1%)	6 (6.3%)	10 (10.5%)	18 (18.9%)	16 (16.8%)
11	57 (60%)	72 (75.8%)	29 (30.5%)	8 (8.4%)	25 (26.3%)	11 (11.6%)
12	29 (30.5%)	54 (56.8%)	43 (45.3%)	59 (62.1%)	17 (17.9%)	7 (7.4%)
13	64 (67.4%)	75 (78.9%)	42 (44.2%)	55 (57.9%)	11 (11.6%)	19 (20%)
14	29 (30.5%)	6 (6.3%)	12 (12.6%)	41 (43.2%)	29 (30.5%)	3 (3.2%)
15	36 (37.9%)	19 (20%)	14 (14.7%)	35 (36.8%)	22 (23.2%)	47 (49.5%)
16	43 (45.3%)	37 (38.9%)	4 (4.2%)	0 (0%)	9 (12%)	38 (40%)
17	42 (44.2%)	31 (32.6%)	31 (32.6%)	12 (12.6%)	19 (20%)	23 (24.2%)
18	70 (73.7%)	20 (21.1%)	8 (8.4%)	31 (32.6%)	35 (36.8%)	0 (0%)
19	38 (40%)	8 (8.4%)	23 (24.2%)	1 (1.1%)	22 (23.2%)	13 (13.7%)
20	20 (21.1%)	12 (12.6%)	26 (27.4%)	12 (12.6%)	30 (31.6%)	30 (31.6%)
21	44 (46.3%)	28 (29.5%)	5 (5.2%)	0 (0%)	18 (18.9%)	5 (5.2%)
22	47 (49.5%)	56 (58.9%)	44 (46.3%)	6 (6.3%)	31 (32.6%)	66 (69.5%)
23	38 (40%)	47 (49.5%)	32 (33.7%)	29 (30.5%)	18 (18.9%)	12 (12.6%)
24	24 (25.3%)	1 (1.1%)	36 (37.9%)	30 (31.6%)	28 (29.5%)	32 (33.7%)
25	22 (23.2%)	4 (4.2%)	25 (26.3%)	11 (11.6%)	25 (26.3%)	7 (7.4%)

**Table 10:** Total amount of correct and close-to-correct (±1) ratings.

Category	T1	T2	*p*-Value (Chi Square Test)
Synovitis	911 (38.4%)	815 (34.3%)	0.004 **
Adhesion	618 (26%)	684 (28.8%)	0.003 **
Deg. Changes	552 (23.2%)	546 (23%)	0.84
Total	2081 (29.2%)	1979 (27.8%)	0.06 (n.s.)

** *p*-Value < 0.005.

**Table 11:** Cases with significant differences between students and master examiners’ ratings for the students’ cohort T1 and T2.

	Synovitis	Adhesion	Degenerative Changes
T1 N/25 (%)	6 (24%)	8 (32%)	5 (20%)
T2 N/25 (%) (*p*-value) Chi Square test	13 (52%) 0.04 *	10 (40%) 0.56 (n.s.)	15 (60%) 0.0004 ***

* *p*-Value < 0.05; *** *p*-Value < 0.0005.

**Table 12:** Fleiss’ Kappa (κ) for both rounds of data acquisition.

	Synovitis Kappa (κ)	Adhesion Kappa (κ)	Degenerative Changes Kappa (κ)
Students at T1	0.06	0.03	0.01
Students at T2 Master examiners	0.1 0.58	0.07 0.50	0.03 0.58

**Table 13:** *p*-values for significant differences between Fleiss’ Kappa (κ) values calculated with the two-sample Z-test.

	Synovitis *p*-Value	Adhesion *p*-Value	Degenerative Changes *p*-Value
Students T1 vs. T2	0.79	0.79	0.91
T1 vs. master Examiners T2 vs. master examiners	0.0003 *** 0.0008 ***	0.0009 *** 0.003 **	0.00007 *** 0.0001 ***

** *p*-Value < 0.005; *** *p*-Value < 0.0005.

## Data Availability

The data presented in this study are available on request from the corresponding author.

## Supplementary Materials

The following supporting information can be downloaded at: https://www.mdpi.com/article/10.3390/jcm13143995/s1, Video S1: Example Video for Temporomandibular Joint Arthroscopy.

## Abbreviations

The following abbreviations are used in this manuscript:

TMDs	Temporomandibular Disorders
TMJ	Temporomandibular joint
TMDs-SevS	Temporomandibular Disorder Severity Score
MRI	Magnetic Resonance Imaging
p	Prediction score
SD	Standard deviation
OMF	Oral maxillofacial
OMFS	oral maxillofacial surgery
i.e.,	id est

## References

[1] Kapos F.P., Exposto F.G., Oyarzo J.F., Durham J. (2020). Temporomandibular Disorders: A Review of Current Concepts in Aetiology, Diagnosis and Management. Oral Surg..

[2] Klasser G.D., Goulet J.-P., De Laat A., Manfredini D., Farah C.S., Balasubramaniam R., McCullough M.J. (2018). Classification of Orofacial Pain. Contemporary Oral Medicine.

[3] Chantaracherd P., John M.T., Hodges J.S., Schiffman E.L. (2015). Temporomandibular Joint Disorders’ Impact on Pain, Function, and Disability. J. Dent. Res..

[4] Gauer R., Semidey M.J. (2015). Diagnosis and Treatment of Temporomandibular Disorders. Am. Fam. Physician.

[5] González-González A.M., Herrero A.J. (2024). A Systematic Review of Temporomandibular Disorder Diagnostic Methods. Cranio.

[6] Wieckiewicz M., Boening K., Wiland P., Shiau Y.-Y., Paradowska-Stolarz A. (2015). Reported Concepts for the Treatment Modalities and Pain Management of Temporomandibular Disorders. J. Headache Pain.

[7] Murakami K., Clark G.T. (1993). Diagnosis of Intracapsular Pathology Associated with Temporomandibular Joint Disorders. Adv. Dent. Res..

[8] Liu F., Steinkeler A. (2013). Epidemiology, Diagnosis, and Treatment of Temporomandibular Disorders. Dent. Clin. N. Am..

[9] Garstka A.A., Kozowska L., Kijak K., Brzózka M., Gronwald H., Skomro P., Lietz-Kijak D. (2023). Accurate Diagnosis and Treatment of Painful Temporomandibular Disorders: A Literature Review Supplemented by Own Clinical Experience. Pain Res. Manag..

[10] Manfredini D., Ahlberg J., Aarab G., Bender S., Bracci A., Cistulli P.A., Conti P.C., De Leeuw R., Durham J., Emodi-Perlman A. (2024). Standardised Tool for the Assessment of Bruxism. J. Oral Rehabil..

[11] Zieliński G., Pająk-Zielińska B., Ginszt M. (2024). A Meta-Analysis of the Global Prevalence of Temporomandibular Disorders. J. Clin. Med..

[12] Kmeid E., Nacouzi M., Hallit S., Rohayem Z. (2020). Prevalence of Temporomandibular Joint Disorder in the Lebanese Population, and Its Association with Depression, Anxiety, and Stress. Head Face Med..

[13] Derwich M., Gottesman L., Urbanska K., Pawlowska E. (2022). Craniovertebral and Craniomandibular Changes in Patients with Temporomandibular Joint Disorders after Physiotherapy Combined with Occlusal Splint Therapy: A Prospective Case Control Study. Medicina.

[14] Onishi M. (1975). Arthroscopy of the temporomandibular joint (author’s transl). Kokubyo Gakkai Zasshi.

[15] Wroclawski C., Mediratta J.K., Fillmore W.J. (2023). Recent Advances in Temporomandibular Joint Surgery. Medicina.

[16] Al-Moraissi E.A., Wolford L.M., Ellis E., Neff A. (2020). The Hierarchy of Different Treatments for Arthrogenous Temporomandibular Disorders: A Network Meta-Analysis of Randomized Clinical Trials. J. Craniomaxillofac. Surg..

[17] de Barros R.C.M., Kato B.K., Sgarbi A.L.G., Tonelli H., Hojaij F.C. (2024). Use of a Smartphone Platform for Temporomandibular Joint Arthroscopy. Int. J. Oral Maxillofac. Surg..

[18] Gutiérrez I.Q., Sábado-Bundó H., Gay-Escoda C. (2022). Intraarticular Injections of Platelet Rich Plasma and Plasma Rich in Growth Factors with Arthrocenthesis or Arthroscopy in the Treatment of Temporomandibular Joint Disorders: A Systematic Review. J. Stomatol. Oral Maxillofac. Surg..

[19] Hakim M.A., Christensen B., Ahn D.Y., McCain J.P. (2020). Correlation of Arthroscopic and Histologic Findings in Synovial Membrane Disease of the Temporomandibular Joint. J. Oral Maxillofac. Surg..

[20] Segami N., Nishimura M., Kaneyama K., Miyamaru M., Sato J., Murakami K.I. (2001). Does Joint Effusion on T2 Magnetic Resonance Images Reflect Synovitis? Comparison of Arthroscopic Findings in Internal Derangements of the Temporomandibular Joint. Oral Surg. Oral Med. Oral Pathol. Oral Radiol. Endod..

[21] Goethe-Zertifikat C1|Goethe-Institut Germany. https://www.goethe.de/ins/de/en/prf/prf/gzc1.html.

[22] Faul F., Erdfelder E., Buchner A., Lang A.-G. (2009). Statistical Power Analyses Using G*Power 3.1: Tests for Correlation and Regression Analyses. Behav. Res. Methods.

[23] Landis J.R., Koch G.G. (1977). The Measurement of Observer Agreement for Categorical Data. Biometrics.

[24] Kim H.-Y. (2017). Statistical Notes for Clinical Researchers: Chi-Squared Test and Fisher’s Exact Test. Restor. Dent. Endod..

[25] Winters R., Winters A., Amedee R.G. (2010). Statistics: A Brief Overview. Ochsner J..

[26] Monje Gil F., Hernandez Vila C., Moyano Cuevas J.L., Lyra M., Pagador J.B., Sanchez Margallo F.M. (2016). Validation of a Simulator for Temporomandibular Joint Arthroscopy. Int. J. Oral Maxillofac. Surg..

[27] Hohn E.A., Brooks A.G., Leasure J., Camisa W., van Warmerdam J., Kondrashov D., Montgomery W., McGann W. (2015). Development of a Surgical Skills Curriculum for the Training and Assessment of Manual Skills in Orthopedic Surgical Residents. J. Surg. Educ..

[28] Aston S., Negen J., Nardini M., Beierholm U. (2022). Central Tendency Biases Must Be Accounted for to Consistently Capture Bayesian Cue Combination in Continuous Response Data. Behav. Res. Methods.

[29] Tran C., Ghahreman K., Huppa C., Gallagher J.E. (2022). Management of Temporomandibular Disorders: A Rapid Review of Systematic Reviews and Guidelines. Int. J. Oral Maxillofac. Surg..

[30] Wadhokar O.C., Patil D.S. (2022). Current Trends in the Management of Temporomandibular Joint Dysfunction: A Review. Cureus.

[31] González-Sánchez B., García Monterey P., Ramírez-Durán M.D.V., Garrido-Ardila E.M., Rodríguez-Mansilla J., Jiménez-Palomares M. (2023). Temporomandibular Joint Dysfunctions: A Systematic Review of Treatment Approaches. J. Clin. Med..

[32] Emmons B.R., Christoforetti J.J., Matsuda D.K., Wolff A.B., Salvo J.P., Martin R., Carreira D.S. (2021). Arthroscopic Classification of Intra-Articular Hip Pathology Demonstrates at Best Moderate Interrater Reliability. Knee Surg. Sports Traumatol. Arthrosc..

[33] Amenabar T., Piriz J., Mella C., Hetaimish B.M., O’Donnell J. (2015). Reliability of 3 Different Arthroscopic Classifications for Chondral Damage of the Acetabulum. Arthroscopy.

[34] Schiffman E., Ohrbach R., Truelove E., Look J., Anderson G., Goulet J.-P., List T., Svensson P., Gonzalez Y., Lobbezoo F. (2014). Diagnostic Criteria for Temporomandibular Disorders (DC/TMD) for Clinical and Research Applications: Recommendations of the International RDC/TMD Consortium Network and Orofacial Pain Special Interest Group. J. Oral Facial Pain Headache.

[35] Wilkes C.H. (1989). Internal Derangements of the Temporomandibular Joint. Pathological Variations. Arch. Otolaryngol. Head Neck Surg..

[36] Ernberg M. (2017). The Role of Molecular Pain Biomarkers in Temporomandibular Joint Internal Derangement. J. Oral Rehabil..

[37] Ibi M. (2019). Inflammation and Temporomandibular Joint Derangement. Biol. Pharm. Bull..

